# Glances at the Hospitals

**Published:** 1905-11-18

**Authors:** 


### GLANCES AT THE HOSPITALS.

### LIVERPOOL HOSPITAL FOR CANCER AND SKIN DISEASES.

The receipts amounted to £1,413 and the expenditure to £1,350, but a sum of £118 was due to bankers and to tradesmen, so that the year closed with a small debit balance. The out-door patients must have been numerous as the average attendances reached 110 a day. This number is in excess of that of the previous year; and, as the committee say, "clearly demonstrates the great advantage the Hospital is to the number of people who cannot afford to pay for medical advice." The in-patients numbered 149, and we are informed that there were many difficult cases which were successfully dealt with. Medical tables are entirely and unaccountably absent; but the body of the report tells us that the *x*-rays and Finsen light have been used in 268 cases of lupus and cancer; and that about 8 per cent. were practically cured, and that a considerable number of others were decidedly improved. Among the cures was one of scirrhus, which had been previously announced to be hopeless, and in which the growth had made considerable progress. At the time of the report "the patient was free from pain, able to attend to her work, and there was no symptom of any re-formation of the growth." The Committee have had to expend £300 in refurnishing the operating theatre, and in providing new beds. They appeal to the public for funds to enable them to meet this necessary expense, which funds we hope will be forthcoming. Private patients are admitted at £2 2s. a week, or in some cases as low as £1 1s.

### LEICESTER INFIRMARY (Medical Report).

On January 1 this infirmary contained 180 patients, 2,717 were admitted during the year, making a total of 2,897 under treatment. Of these 2,388 were discharged either cured or relieved, 132 for other reasons, 191 died, and 186 remained in the wards at the end of December. The average stay in the house of the medical cases was nearly twenty-eight days, and of the surgical cases nineteen days. The statistical tables are prepared with much clearness and give sufficient information in all cases. For instance, we note that of thirty cases of lobar pneumonia, twenty-five were cured, three died, and one remained under treatment. There were seven cases of diphtheria, all of whom recovered. Of seven cases of enteric fever, six recovered and one was under treatment. Among other things the table of operations shows that there were forty-seven amputations with two deaths. The above included fifteen amputations of the thigh and both the fatal cases were among these. Fifteen cases of ovariotomy gave two deaths. The operation for perforated gastric ulcer was performed eleven times with four recoveries and seven deaths. The total number of major operations performed during the year was 1,604, and of minor operations 1,372. Anæsthetics were administered 2,976 times. In 1,304 cases chloroform was the agent; in 1,028, ether; in 398, nitrous oxide; in 157, ether and chloroform; in 39, nitrous oxide and somnoform; in 20, chloroform and A.C.E.; in 13, nitrous oxide and ethyl chloride; in 3, A.C.E.; in 3, ethyl chloride; and in 3, ether and ethyl chloride.

